# Development and Validation of the Systems Thinking in Food and Agricultural Science Scale

**DOI:** 10.3390/foods15213933

**Published:** 2026-11-03

**Authors:** Kevan W. Lamm, Haoming Fan, Alexa J. Lamm, Catherine E. Sanders, Pedro E. Urriola, Derrick J. Coble, Katy Guthrie

**Affiliations:** 1 Department of Agricultural Leadership, Education & Communication, University of Georgia, Athens, GA 30602, USA; haomingfan@uga.edu (H.F.); alamm@uga.edu (A.J.L.); 2 Department of Agricultural and Human Sciences, North Carolina State University, Raleigh, NC 27695, USA; ecdobbin@ncsu.edu; 3 Department of Animal Science, University of Minnesota, St. Paul, MN 55108, USA; urrio001@umn.edu; 4 Department of Animal Sciences, North Carolina Agricultural and Technical State University, Greensboro, NC 27411, USA; djcoble@ncat.edu; 5 Department of Agronomy and Plant Genetics, University of Minnesota, St. Paul, MN 55108, USA; guthr103@umn.edu

**Keywords:** systems thinking, food systems, scale development, competency assessment, agricultural education

## Abstract

Systems thinking is widely recognized as a foundational competency for solving complex problems in food and agricultural systems. However, existing studies lack validated, domain-specific instruments to evaluate learners’ systems thinking. This study addresses this gap by providing initial validity evidence for the Systems Thinking in Food and Agricultural Science (STFAS) scale with a four-stage design: (1) item generation from the literature and a 17-member expert panel, (2) content-validity review by 10 systems-thinking experts, (3) cognitive interviews with 8 representatives of the intended population, and (4) psychometric testing of the revised 23-item pool with a U.S. general-public sample (*N* = 993). The cleaned data were randomly split into two halves for exploratory (*n* = 495) and confirmatory (*n* = 498) factor analysis. Exploratory factor analysis supported a 15-item, two-factor solution: (1) Systems Structure and Dynamics (8 items) and (2) Systems Mindset and Interpretation (7 items). Confirmatory factor analysis indicated acceptable fit (CFI = 0.935; TLI = 0.924; RMSEA = 0.068; SRMR = 0.052) with strong internal consistency (α = 0.86 to 0.91). STFAS scores were positively associated with a domain-general systems thinking scale (convergent validity); the two factors were empirically distinct (discriminant validity), and scores were negatively associated with socially desirable responding, indicating no inflation by favorable self-presentation. The STFAS can support research on stakeholder decision-making in agri-food system transformation.

## 1. Introduction

Agriculture is a cornerstone of the global economy and is facing unprecedented challenges to feed a global population predicted to reach 10 billion by 2050, while holding the responsibility to reduce environmental degradation, adapt to extreme weather events, and ensure access to safe and nutritious food [1,2]. Specifically, the food system connects production, processing, distribution, retailing, consumption, and disposal of food, which involves a wide range of actors, including producers, processors, retailers, policymakers, and consumers [3,4,5]. As a result, the challenges facing agriculture are increasingly recognized as broader system challenges, in which outcomes from food security, nutrition, environmental sustainability, and farm livelihoods are shaped across the entire system rather than at the individual farm-level [6,7,8]. To address these challenges, researchers and professionals must look beyond traditional, narrow disciplinary-based analysis and move toward a systems approach to address complex challenges [9,10]. Agricultural and food system challenges are typically embedded across a range of social, ecological, economic, local, and global levels, where an intervention in one part of a system may trigger cascading unintended consequences in other areas [11,12,13,14]. As a result, food and agricultural systems are often considered complex adaptive systems [15,16], characterized by various interacting components, nonlinear feedback, time delays, and emergent behaviors [14,15,17,18].

Systems thinking has been recognized as a cognitive orientation to conceptualize complex adaptive systems by focusing on the relationships, feedback structures, and dynamics that connect the various components and monitor systemic behavior over time [12,19,20]. For example, in the context of agricultural sustainability, systems thinking has been identified as a foundational competency that facilitates the ability to analyze complex problems holistically and plan durable interventions [21,22,23]. Previous scholars have suggested that systems thinking serves as a keystone of professional preparation for agriculture and food science learners by guiding them to forecast the trade-offs across production systems, the environment, nutrition, and society [24,25]. In the context of the food system, systems thinking may provide a useful cognitive approach to assist agricultural and food system professionals in translating complex scientific and systemic knowledge for a range of stakeholders, informing how practitioners and decision makers frame and address food-system-related problems [26,27].

Despite the importance associated with systems thinking in the food system, a validated scale assessing systems thinking is notably absent, particularly when compared to domains such as healthcare, engineering, and business [28,29,30]. Existing assessments of systems thinking often rely on concept maps, causal loop diagrams, or general self-report instruments used to elicit learners’ reasoning, but these approaches potentially neglect unique disciplinary features of agricultural and food systems [31,32,33]. In agriculture, fostering systems thinking has been proposed to involve more than introducing terminologies or conceptual diagrams [34]. Specifically, learners must reason about the interacting biological, economic, environmental, and social components of food systems [33,35,36]. Additionally, scholars have suggested that agriculture and food science learners need structured opportunities to practice systems-oriented reasoning and test consequences of their decisions [37,38]. However, a measure to assess systems-thinking development in this domain has been unavailable. To address this gap, this study reports the initial development of the Systems Thinking in Food and Agricultural Science (STFAS) scale, a self-report instrument to measure systems-thinking reasoning. Beyond formal instruction, systems thinking also shapes how consumers, producers, and other stakeholders reason about and respond to change across the food system, which makes a measure of this reasoning useful for studying the behavioral dimension of agri-food system transformation.

### 1.1. Systems Thinking: Origins and Core Constructs

The intellectual origins of systems thinking can be linked to general systems theory [39] and systems dynamics theory [14], both intended to investigate the interconnections between elements and forecast long-term behavior patterns [40,41]. The notion of systems theory has received mainstream educational recognition largely through Senge’s work [20], which advocates systems thinking as the fifth discipline that integrates the various disciplines within learning organizations. A later foundational work and synthesis was produced by Meadows [12], who described four basic components of systems: (1) the behavior of stocks and flows, (2) balancing and reinforcing feedback loops, (3) delays, and (4) the identification of leverage points [12]. Although a precise definition of systems thinking may vary across disciplines, the fundamental aspects tend to converge into a common set of features, including the prioritization of interconnections and relationships beyond isolated parts, recognition of feedback and circular causality, awareness of dynamic behavior, delays, nonlinearity, and emergence, and the idea of mental models that shape how individuals interpret a system [12,19,20].

Within the literature, numerous systems thinking frameworks have been constructed to capture learners’ systems thinking and development. For example, Cabrera and colleagues [42] distilled systems thinking into four underlying cognitive rules, including (1) recognizing distinctions, (2) organizing ideas into part-whole systems, (3) noticing relationships, and (4) being aware of multiple perspectives [42]. Other scholars have described systems thinking as a set of actionable reasoning habits, involving surfacing, testing, and revising assumptions and mental models [43,44,45]. Additionally, in the context of science and environmental education, systems thinking has been proposed as a hierarchical model that has progressively developed from the ability to identify a system’s components toward the ability to understand its cyclic and hidden dimensions [46]. Collectively, these frameworks present systems thinking as comprising multiple, distinguishable facets rather than a single attribute [47].

### 1.2. Systems Thinking in Food and Agriculture

In the context of food and agriculture systems, farmers and policymakers often encounter problems with challenges in defining, observing, and measuring inputs and interactions accurately [34]. Unlike technical problems, which can be solved through more linear forms of reasoning [48], problems in agriculture and food sciences often emerge from the interaction of multiple system components [48,49]. For example, a food system may involve biological, environmental, technological, economic, social, and policy-related interactions in producing, processing, distributing, consuming, and disposing of food [50,51]. As a result, decisions at one node can cause a ripple effect and a series of unpredictable consequences in areas such as water quality, economic viability, food prices, and dietary consumption patterns, resulting in the so-called butterfly effect and unintended emergent behaviors [52,53,54]. The complex nature of food and agriculture system challenges has been characterized as wicked problems that lack definitive formulations or technical solutions [55]. As a result, decisions regarding the agri-food system have been recommended to actively include a holistic perspective and use systems thinking skills to understand interconnections, feedback loops, delays, and nonlinear relationships [18,34,56].

The development of systems thinking in agricultural and food systems education has received increasing recognition. In the context of sustainable food systems education, systems thinking has been identified as a central pedagogical approach and an important tool to encourage experiential learning [25]. Traditionally, many programs in agricultural education have advocated building curricula, research methods, and Extension strategies based on a systemic paradigm [24,57]. Additionally, in the context of Extension and outreach, scholars have recommended developing competencies beyond technical and narrowly defined expertise to engage with the broader agricultural systems, recognize the interconnections between resources, and accelerate technology adoption [26,27].

### 1.3. Literature Review: Measuring Systems Thinking

Despite the well-recognized and acknowledged importance of systems thinking in agricultural and food science education [36], a domain-specific systems thinking competency requires a valid and reliable measure before it can be effectively evaluated or studied [58]. The following section examines the theoretical foundation for measuring systems thinking and reviews existing instruments, establishing why a domain-specific measure is needed.

#### 1.3.1. Theoretical Foundations: Systems Thinking

Contemporary scholarship frames systems thinking as an integrated set of knowledge, skills, and dispositions, allowing people to solve complex problems through a series of logical reasoning [31]. In the context of food science, systems thinking is generally identified as foundational, underpinning the analytic, anticipatory, and strategic capacities on which other competencies depend [21,22,59]. Competency is defined as “a bundle of skills, knowledge and abilities that are required to deal effectively with complex and dynamic non-routine situations in different domains” [60] (p. 42), involving contextualized reasoning tendencies rather than abstract self-evaluations [61]. The multidimensional nature of systems thinking requires measurements that represent distinguishable facets and various reasoning abilities rather than a single dichotomous classification [46]. As a result, systems thinking often spans beyond technical evaluations and covers a range of topics, including tracing feedback, interdependencies, and dynamic behavior, interpreting ambiguous information, and anticipating systemic outcomes [31,42].

#### 1.3.2. Existing Instruments for Measuring Systems Thinking

Despite numerous instruments established to measure systems thinking, few address the agriculture and food domain. For example, in the healthcare context, the Systems Thinking Scale offers a brief self-report measure of systems thinking in clinical and quality-improvement settings [28,45]. In the engineering and systems science context, instruments have been established to measure individuals’ systems thinking capacity and predisposition to systems thinking categories with additional cognitive and affective measures for engineering education [29,44,62]. In the management education field, systems thinking self-report measurement has been used to assess systems thinking among business students [30]. Lastly, in the sustainability and environmental science field, systems thinking measurements have typically focused on specific topics, such as earth systems and climate change [46,63].

In addition to self-report scales, researchers and educators often assess systems thinking by evaluating learners’ performance by solving intentionally ill-structured problems, using methods such as drawing causal-loop diagrams and concept maps and eliciting mental models [31,32,33,43]. An additional approach includes the performance check process [31]. The process provides personalized systems thinking assessment and clear feedback for improvement, but it is time-consuming and largely relies on the evaluators’ teaching experiences [64]. As a result, in agriculture and food contexts, systems thinking has generally been examined as a pedagogical aim through domain-specific conceptual mapping rather than assessed using a validated measurement instrument [33,36].

The complexity of the agricultural and food systems domain has presented difficulties in developing a brief, validated, and contextually specific self-report instrument to measure systems thinking [28]. Specifically, work within food and agricultural systems requires contextualized knowledge of components, feedback relationships, interdependencies, and trade-offs, which can differ substantially from clinical, engineering, or business contexts [34,65]. As a result, instruments developed for other domains may not adequately capture the complexity in food and agricultural systems [34,66]. Taken together, these limitations establish the need for a brief, domain-specific measure developed and validated for food and agricultural systems, which is the aim of the present study.

### 1.4. Purpose and Research Objectives

The purpose of this study was to develop and provide initial validity evidence for the Systems Thinking in Food and Agricultural Science (STFAS) scale. This study is guided by contemporary validity theory, in which validity is treated as a unitary judgment supported by multiple sources of validity evidence [61], namely content, response process, internal structure, and relations with other variables. This study also aims to answer the following questions:

(1) What dimensional structure of systems-thinking reasoning was represented by the STFAS item pool?
(2) Do STFAS scores exhibit adequate reliability?
(3) Do STFAS scores demonstrate convergent and discriminant validity in relation to external measures?

## 2. Materials and Methods

Data were collected from U.S. adults aged 18 and older. Participants were recruited through a non-probability opt-in online panel and completed the instrument in Qualtrics. Predetermined quotas for geographic region, age, gender, race, and ethnicity were used to align the sample with the U.S. adult target population [67]. Of the 1000 adults recruited, 993 were retained after screening for attention-check failures, straight-line responding, and implausibly short completion times, with no item-level missing data.

To conduct the factor analysis, the cleaned data set was randomly divided into two approximately equal halves [68], one used for exploratory factor analysis (EFA; *n* = 495) and the other for confirmatory factor analysis (CFA; *n* = 498). The two subsamples did not differ on measured demographic characteristics (all |t| < 1.2; all χ^2^ *p* > 0.20), supporting the equivalence assumption for the cross-validation design [68].

### 2.1. Scale Development Procedure

The STFAS was developed through a four-phase process based on scale development practice and the sources of validity evidence described in the Standards for Educational and Psychological Testing [61,69,70,71]. In Phase 1 (item generation), the research team analyzed the existing literature related to systems thinking and extracted two primary themes that are complementary dimensions: (1) technical in nature (knowledge of system components, feedback structures, and dynamic behavior) or (2) conceptual in nature (interpretive habits such as anticipating system behavior and weighing multiple perspectives). After identifying the two primary themes, a panel of 17 invited experts was surveyed for item drafting, eliciting an initial pool of 27 items. In Phase 2 (content validity), 10 systems-thinking experts evaluated the pool for relevance, clarity, and representativeness, reducing the 27 items to 23. Items with low expert-rated relevance were removed. For example, “I notice how my brand loyalty encourages stores to stock more of that brand” was dropped because it reflected brand or marketing familiarity more than systems reasoning, and “I notice how holiday promotions tend to lead to higher snack prices later in the season” was dropped because its reference to two separate time periods was unclear and redundant with other price items. Other items were revised rather than removed; for example, an item whose stakeholder referent was judged too general became “I compare beekeepers’, crop growers’, and scientists’ opinions on pesticide bans.” In Phase 3 (response process validity), 8 participants representative of the intended population completed the items and described their reasoning as they responded, providing response-process evidence on how respondents understood and answered the items [72]. The feedback was organized using the four cognitive stages of survey response, comprehension, retrieval, judgment, and response [73], and indicated occasional comprehension and retrieval difficulties that informed wording revisions. For example, an item that some respondents could not answer because they lacked experience with its example, energy-drink consumption, was revised to a more general comparison of dietitians’ and influencers’ views on diet advice. In Phase 4 (internal and external structure validity), the revised 23-item pool was tested using a sample (*N* = 993) that was subjected to split-sample analysis, including EFA and CFA, to test reliability and external validity, resulting in a retained 15-item scale composed of two factors.

### 2.2. Instrumentation

The retained STFAS scale represented a domain-specific measure of systems-thinking capacity in food and agriculture. The final instrument comprised 15 items with two correlated factors, informed by the technical and conceptual themes that guided item generation, including (1) Systems Structure and Dynamics (8 items), reflecting attention to system components, feedback, and dynamics, and (2) Systems Mindset and Interpretation (7 items), reflecting interpretive reasoning, including anticipating system behavior, weighing multiple perspectives, and interpreting information. All items share the stem, “When I analyze a problem in the food or agricultural system…,” and are rated on a 5-point Likert-type metric ranging from 1 (strongly disagree) to 5 (strongly agree). Because the technical and conceptual themes guided item generation but did not empirically map one-to-one onto the emergent factors, each factor is interpreted on its own terms; the two factors are abbreviated Structure & Dynamics and Mindset & Interpretation in the tables that follow.

Conceptually, the two factors differ in the object of reasoning. Systems Structure and Dynamics concerns the structural and causal mechanisms of the food system, including multi-factor causation and trade-offs, whereas Systems Mindset and Interpretation concerns its emergent and interpretive aspects, including anticipating behavioral responses, weighing multiple stakeholder perspectives, and interpreting ambiguous information.

### 2.3. Comparison Measures

Two established scales were administered alongside the STFAS to test external validity. Specifically, convergent evidence was obtained using a five-item version of the Dolansky Systems Thinking Scale (STS) [28], a domain-general measure of systems thinking rated on a 5-point Likert-type scale. To check for favorable self-presentation bias, the short-form (three-item) measure of socially desirable responding (SDR) [74] was administered as a response-bias check. It should be noted that the original version of SDR included five items; however, two reverse-worded items were removed upon subsequent analysis because of weak item-total correlations. The remaining three items were retained (α = 0.80).

### 2.4. Data Analysis

Exploratory factor analysis was conducted in SPSS 29. Confirmatory factor analysis, reliability, and validity analyses were conducted in Stata 17, with a statistical significance level of α = 0.05. Consistent with the cross-validation design, the factor structure was first derived through EFA in one random half of the sample and then independently tested through CFA in the hold-out half.

#### 2.4.1. Exploratory Factor Analysis

The factorability of the item correlation matrix was assessed using the Kaiser-Meyer-Olkin (KMO) measure of sampling adequacy [75] and Bartlett’s test of sphericity. Factors were extracted through principal-axis factoring with promax (oblique) rotation (κ = 4), and the number of factors was determined by parallel analysis [76] and inspection of the scree plot. Items were retained when they satisfied all of the following criteria: a primary loading of at least 0.45, a difference of at least 0.20 between the primary and next-highest loading, a communality (h^2^) of at least 0.30, and conceptual distinctiveness [77,78,79]. Item reduction proceeded iteratively across three analytic passes. In the first pass, two items were removed for structural problems, namely one item that cross-loaded (≈0.30/0.44) without a dominant factor and one item that loaded weakly on both factors (≈0.40/0.30), resulting in 21 items. For example, “I see that national policy changes affect cereal prices” cross-loaded across both factors without a dominant loading and was removed in the first pass. In the second pass, a more stringent primary-loading threshold of 0.55 was applied, and five items below this threshold were removed, leaving 16 items. In the third pass, one additional item was removed on the basis of a parsimony and redundancy judgment: of the two weakest remaining items (I predict that when meat prices pass a tipping point, shoppers will switch to non-meat protein alternatives ≈ 0.55 and I notice banning trans-fats results in food makers adding more sugar instead ≈0.52), the trans-fats item was dropped and the meat prices item retained because it was more construct-distinct and the content of the trans-fats item was already represented by other items. As a result, 15 items were included in the final scale. The 15 items were associated with two emergent factors named Systems Structure and Dynamics (8 items) and Systems Mindset and Interpretation (7 items). The retained 15 items preserved the breadth of the construct, representing both dimensions and the range of systems-thinking principles that informed item generation.

#### 2.4.2. Confirmatory Factor Analysis

A two-factor model, with each item loading on a single factor and error terms left uncorrelated, was estimated by maximum likelihood in the hold-out sample. Model fit was evaluated against the criteria recommended by Hu and Bentler [80], namely CFI and TLI ≥ 0.90, RMSEA ≤ 0.08 (within the 90% confidence interval), and SRMR ≤ 0.08, and the two-factor model was compared with a competing one-factor model.

#### 2.4.3. Reliability and Validity

Internal consistency reliability was estimated using Cronbach’s alpha, McDonald’s omega, and composite reliability. Convergent validity was assessed through the average variance extracted (AVE ≥ 0.50) and the correlation and regression of STFAS scores on the Dolansky STS. Discriminant validity was evaluated using the Fornell–Larcker criterion, comparing the square root of each factor’s AVE with the inter-factor correlation [81]. Separately, as a check on response bias rather than discriminant validity, the association between STFAS scores and socially desirable responding was examined through correlation and regression.

## 3. Results

### 3.1. Descriptive Statistics and Reliability

Factor and total scores were computed as the mean of the constituent items on the original 5-point Likert-type scale. Descriptive statistics and reliability results (Table 1) indicated that participants, on average, scored higher on Systems Structure and Dynamics (M = 3.75, SD = 0.68) than on Systems Mindset and Interpretation (M = 3.23, SD = 0.86), with an overall STFAS mean of 3.51 (SD = 0.68). Internal consistency was strong for both factors as well as the full scale (Cronbach’s α = 0.86, 0.88, and 0.91, respectively). The McDonald’s omega and composite reliability scores fell within acceptable ranges (ω = 0.87 and 0.89; CR = 0.88 and 0.90). Average variance extracted for Systems Mindset and Interpretation was 0.51 and Systems Structure and Dynamics was 0.43, which is below the conventional 0.50 benchmark, indicating that results for this factor should be interpreted with caution [81]. Nonetheless, composite reliability for Systems Structure and Dynamics remained adequate (CR = 0.88), and an AVE below 0.50 accompanied by acceptable composite reliability is commonly interpreted as still supporting convergent validity [81].

### 3.2. Exploratory Factor Analysis (n = 495)

The item correlation matrix of the derivation sample indicated that the data were suitable for factor analysis (KMO = 0.88; Bartlett’s test of sphericity χ^2^(253) = 4954.7, *p* < 0.001). Parallel analysis and inspection of the scree plot converged on a two-factor solution (eigenvalues = 6.08 and 2.98), accounting for 44% of the common variance. The Promax rotation yielded a simple structure with an inter-factor correlation of r = 0.38. The rotated pattern loadings for the final 15 items (Table 2) indicated that primary loadings of Systems Structure and Dynamics ranged from 0.63 to 0.85, and Systems Mindset and Interpretation ranged from 0.55 to 0.85.

### 3.3. Confirmatory Factor Analysis (n = 498)

The two-factor structure was tested in the independent hold-out sample. The model demonstrated an acceptable fit to the data, χ^2^(89) = 292.43, *p* < 0.001; CFI = 0.935; TLI = 0.924; RMSEA = 0.068, 90% CI [0.059, 0.077]; SRMR = 0.052. All standardized factor loadings were statistically significant (*p* < 0.001), ranging from 0.55 to 0.91. The two latent factors were moderately correlated (φ = 0.62). A competing one-factor model provided substantially poorer fit (ΔCFI = −0.09), supporting retention of the two-factor structure over a unidimensional alternative.

### 3.4. Convergent Validity

Results in Table 3 indicated that STFAS scores were positively associated with the Dolansky STS. Specifically, the full STFAS accounted for 47% of the total variance in STS scores (R^2^ = 0.47), with a correlation of 0.76 for Systems Structure and Dynamics and 0.47 for Systems Mindset and Interpretation. The weaker association for Systems Mindset and Interpretation suggests that this factor captures interpretive and perspective-based reasoning that the Dolansky measure does not fully represent.

### 3.5. Discriminant Validity and Response Bias

Discriminant validity was supported by the Fornell–Larcker criterion: the square root of the AVE was 0.66 for Systems Structure and Dynamics and 0.72 for Systems Mindset and Interpretation, both exceeding the latent factor correlation of 0.62, indicating that the two factors capture distinct aspects of the construct [81]. As a separate check on response bias rather than as discriminant evidence, the association between STFAS scores and socially desirable responding was examined. STFAS scores were negatively related to socially desirable responding, with the total score accounting for 20% of the variance in SDR (B = −0.67, R^2^ = 0.20), indicating that higher STFAS scores were not an artifact of favorable self-presentation.

## 4. Discussion

The study developed and provided initial validity evidence for the STFAS, addressing the absence of a domain-specific systems-thinking measure for food and agriculture. The results support a 15-item instrument with correlated dimensions. Specifically, the Systems Structure and Dynamics dimension appears to evaluate respondents’ understanding of how system components, feedback, and dynamics interrelate. The Systems Mindset and Interpretation dimension appears to reflect interpretive reasoning skills, such as anticipating behavior, weighing multiple perspectives, and interpreting information. The two-dimensional structure emerged from EFA results and was confirmed using an independent holdout sample. The two-dimensional structure is consistent with the theoretical premise that systems thinking is multidimensional rather than a single, undifferentiated trait [12,19,42]. This two-dimensional structure mirrors a broader division in the systems-thinking literature, in which research and assessment tend to emphasize either broad conceptual understanding or more technical, structural reasoning about system mechanics [82]. By representing both within a single brief instrument, the STFAS spans a division the field has been encouraged to bridge [82].

The results indicate strong internal consistency, acceptable model fit, and strong associations with external measurements. Specifically, convergent and discriminant validity were supported using the Dolansky STS [28] and Fornell–Larcker criteria [81]. The positive relationship with the Dolansky STS may indicate that the STFAS effectively evaluates systems-thinking reasoning across both Structure and Dynamics and Mindset and Interpretation. Nonetheless, the Dolansky STS is a domain-general instrument scored using a single index without differentiating technical from conceptual reasoning [28]. The results from convergent correlations are consistent with a two-factor design, as STS scores were more strongly associated with Systems Structure and Dynamics (r = 0.76) than with Systems Mindset and Interpretation (r = 0.47). The differences in the correlations indicate that the domain-general Dolansky STS overlaps more strongly with Systems Structure and Dynamics than with Systems Mindset and Interpretation, which captures interpretive reasoning skills. In this way, the STFAS extends existing measurement by distinguishing these two dimensions within a single brief scale in the context of agricultural and food sciences. The Fornell–Larcker results indicate that the two factors are empirically distinguishable, and the negative correlation with socially desirable responding indicates that a favorable self-presentation bias did not inflate STFAS scores. The negative association may also be meaningful rather than only indicating the absence of bias. Because systems thinking entails attention to complexity, interdependence, and uncertainty [12,19], respondents with stronger systems reasoning may be more aware of the limits of simple explanations and correspondingly less inclined to endorse idealized or socially desirable statements, an interpretation consistent with the observed inverse relationship that nonetheless requires confirmation in future work.

The present study’s validation relied on a U.S. general-public sample recruited with quotas matched to U.S. Census demographics. Although systems thinking is frequently framed in professional terms, food and agricultural systems are shaped by consumers and the general public as well as by producers, educators, and Extension professionals [83]. The STFAS items were accordingly written to assess reasoning about everyday food-system phenomena, such as grocery prices, food waste, and competing dietary advice, at a level accessible across expertise. Recruiting a demographically diverse sample is consistent with the recommendation that, to extend a new scale’s generalizability, “sample diversification should be considered in terms of data collection, particularly in the psychometric evaluation step” (p. 12) [84]). A heterogeneous, census-matched general-public sample is therefore appropriate for an initial evaluation of the instrument’s dimensional structure and psychometric properties, and it bears directly on how non-specialist stakeholders reason about food-system change. Because demonstrated competency entails contextualized knowledge and skills [60], the present evidence establishes the structure and initial validity of the scale rather than performance among domain professionals, and validation in student, producer, and Extension populations remains a priority for future work.

It is also important to note that the scale’s reference to food and agricultural science uses the term in its broad, system-level sense rather than a narrowly technical one. Under the United States National Agricultural Research, Extension, and Teaching Policy Act [85], the food and agricultural sciences span the physical and social sciences and include nutritional sciences and “the processing, distributing, marketing, and utilization of food and agricultural products” [NARETPA]. Everyday food choices and the reasoning that accompanies them are therefore encompassed by the food and agricultural sciences as formally defined, and the STFAS items, which concern how people reason about the production, pricing, distribution, and consumption of food, fall within that scope.

### 4.1. Contributions to Theory

The STFAS is, to our knowledge, the first food- and agriculture-specific, theoretically grounded measure of systems thinking. Prior instruments have been developed for, and validated in, other domains, such as healthcare, engineering, and business [28,29,30]. The STFAS operationalizes systems thinking in relation to specific components, feedback, and trade-offs within the eco-agri-food system. By representing both structural and interpretive dimensions of systems reasoning in a brief, accessible format, the instrument provides a foundation for cumulative research on how systems thinking reasoning develops in this domain.

### 4.2. Contributions to Practice

The proposed STFAS is a brief, self-report instrument that can support the assessment of systems thinking reasoning in agricultural and food-systems education and Extension. It offers educators and associated agri-food professionals a practical tool to assess individuals’ systems reasoning skills and a measure with initial validity evidence for assessing professional development. Additionally, the STFAS fills a gap in the current agriculture and food science sector that requires domain-specific knowledge of biophysical processes, supply chains, sustainability, and stakeholder relationships [36,57]. The absence of an agriculture- and food-focused systems thinking measurement scale creates consequential validity [33,34] risks when educators and professionals borrow instruments calibrated to other domains, leading to potentially unreliable assessments which do not align with agricultural domains.

### 4.3. Limitations and Future Research

Despite the encouraging findings from the study, there are several limitations which must be addressed. First, as a self-report instrument, the STFAS may be influenced by self-serving bias. Specifically, respondents may overestimate their competency due to overconfidence or favorable self-presentation, or the STFAS may underestimate the participants’ ability to solve scenario-based tasks by failing to capture the nuances in their competence. Despite the negative association between STFAS and SDR scores suggesting that favorable self-presentation did not inflate self-ratings, the result only captures the motivation to respond desirably and cannot detect miscalibrated self-assessment. Therefore, pairing the STFAS with behavioral or scenario-based assessments should be considered to strengthen interpretability and accuracy of the assessment results [31,32]. The self-report competency evaluation provides scalable measurement that can be used as a complement to performance and scenario-based assessment [31,58]. Second, the validation process relied on a general-public sample; future studies should consider cross-validation in alternative populations of interest, including agricultural and food-systems education settings. Third, additional reliability and validity evidence remains to be established, including test–retest reliability and criterion-related validity against external indicators of systems-thinking performance.

Furthermore, measurement invariance was not examined as part of this study. The present study provides preliminary validity evidence and does not compare scores across groups or measurement occasions, for which invariance is a precondition; as Putnick and Bornstein [86] noted, “prior to testing mean differences across groups or measurement occasions […] it is essential to assess the invariance of the construct” (p. 71). Formal tests of configural, metric, and scalar invariance were therefore beyond the scope of this initial validation. Because the STFAS is intended to support between-group comparisons and pre- and post-intervention assessment, establishing invariance across relevant demographic and experiential subgroups is an important next step for future validation.

Additionally, the STFAS items were deliberately grounded in everyday, lived experience and written in simple, familiar language rather than technically precise terms that a general audience might struggle to interpret, consistent with the recommendation to use simple and familiar words, avoiding technical terms and jargon [87]. A trade-off of this accessibility is that several items rely on context-specific, time-bound examples, such as the smoothie-trend and meat-price items, that may date or may not transfer directly across cultural settings. Caution is therefore warranted when adapting the instrument to other cultural or temporal contexts, and such adaptation should include re-examination of item wording and, ideally, re-validation in the target context.

## 5. Conclusions

This study developed and provided initial evidence of validity for the Systems Thinking in Food and Agricultural Science (STFAS) scale, addressing a gap in the literature, specifically a domain-specific measure of systems thinking in food and agriculture. Through the split-sample design, the STFAS emerged as a 15-item instrument with two theoretically coherent and empirically distinct factors with strong internal consistency, acceptable model fit, and supportive convergent and discriminant evidence. Although further validation in alternative populations is recommended, the STFAS represents an initial, theoretically grounded step toward measuring systems thinking reasoning in food and agricultural systems.

## Figures and Tables

**Table 1:** Descriptive statistics and reliability (*n* = 498).

Factor	Items	M	SD	α	ω	CR	AVE
Structure & Dynamics	8	3.75	0.68	0.86	0.87	0.88	0.43
Mindset & Interpretation	7	3.23	0.86	0.88	0.89	0.90	0.51
Overall STFAS	15	3.51	0.68	0.91	—	—	—

Note. α = Cronbach’s alpha; M = the mean of the constituent items on the original 5-point Likert-type scale (possible range: 1–5); ω = McDonald’s omega; CR = composite reliability; AVE = average variance extracted.

**Table 2:** Promax pattern loadings for the final 15 items (*n* = 495).

Item	Structure & Dynamics	Mindset & Interpretation
I notice how rising overseas shipping costs increase grocery prices where I shop.	0.70	—
I recognize when local shortages, not global trends, result in higher egg prices.	0.71	—
I recognize how organic farming methods affect the final price I pay.	0.63	—
I anticipate that drought in another region raises produce prices at my store.	0.73	—
I recognize that there are likely many factors, not one single cause, increasing rates of obesity.	0.85	—
I notice single-serve coffee pods save time but create lots of waste.	0.68	—
I expect that reducing household food waste will require several changes, not one fix.	0.79	—
I recognize that organizations must balance daily operations and long-term goals.	0.66	—
I think about why food-delivery fees spike on rainy Friday nights.	—	0.85
I expect a sudden smoothie trend to sell out frozen Açai fast.	—	0.76
I compare dietitians’ and influencers’ views before judging diet advice.	—	0.77
I compare beekeepers’, crop growers’, and scientists’ opinions on pesticide bans.	—	0.69
I notice when farmers, food processors, and retailers fail to share information about their operations.	—	0.62
I see how social media chefs and environmental groups highlight plant-based meats in different ways.	—	0.61
I predict that when meat prices pass a tipping point, shoppers will switch to non-meat protein alternatives.	—	0.55

Note: Loadings < 0.30 suppressed. Factor correlation r = 0.38.

**Table 3:** Convergent and discriminant validity correlations (*n* = 498).

STFAS Score	r with Dolansky STS	r with SDR
Structure & Dynamics	0.76	−0.24
Mindset & Interpretation	0.47	−0.54
Overall STFAS	0.69	−0.45

Note: For all correlations, *p* < 0.001. Dolansky STS = domain-general systems-thinking measure; SDR = socially desirable responding.

## Data Availability

The datasets presented in this article are not readily available because the data are part of an ongoing study. Requests to access the datasets should be directed to the corresponding author.
